# Profiling metabotropic glutamate receptor 7 expression in Rett syndrome: consequences for pharmacotherapy

**DOI:** 10.1016/j.neuroscience.2026.01.040

**Published:** 2026-01-30

**Authors:** Sheryl Anne D. Vermudez, Geanne A. Freitas, Mackenzie Smith, Rocco G. Gogliotti, Colleen M. Niswender

**Affiliations:** a Department of Pharmacology and Warren Center for Neuroscience Drug Discovery, Vanderbilt University, Nashville, TN 37232, USA; b Department of Molecular Pharmacology and Neuroscience, Loyola University Chicago, IL 60660, USA; c Vanderbilt Institute of Chemical Biology, Vanderbilt University, Nashville, TN 37232, USA; d Vanderbilt Brain Institute, Vanderbilt University, Nashville, TN 37232, USA; e Vanderbilt Kennedy Center, Vanderbilt University Medical Center, Nashville, TN 37232, USA

**Keywords:** Metabotropic glutamate receptors, mGlu_7_, Rett Syndrome, *MECP2*

## Abstract

Rett syndrome (RTT) is caused by mutations in the X-linked methyl-CpG binding protein 2 (MeCP2) transcription factor. RTT patients undergo a developmental regression between 6–18 months of age, resulting in the presentation of symptoms including repetitive behaviors, seizures, autistic-like features, and apneas. We have reported that levels of metabotropic glutamate receptor 7 (mGlu_7_) are significantly decreased in brain samples from RTT patients carrying truncation mutations in the *MECP2* gene. Additionally, we have identified decreases in *Mecp2*^+*/−*^ mice and demonstrated that administration of a positive allosteric modulator (PAM) with activity at mGlu_7_ corrected deficits in cognitive, social, and respiratory domains. Here, we expanded our studies to a larger cohort of RTT samples covering a range of mutations and evaluated expression of the three widely expressed group III mGlu receptors (mGlu_4,7 and 8_). We found significant decreases in mGlu_7_, but not mGlu_4_ or mGlu_8_, mRNA expression across this larger cohort; additionally, we identified a previously unknown correlation in the expression of mGlu_4_ and mGlu_8_ in human brain samples. Stratification of RTT patients into those with classically severe versus mild *MECP2* pathogenic mutations revealed statistically significant decreases in mGlu_7_ expression only in patients with mutations associated with severe symptoms. To establish whether target disruption is required for efficacy, we administered the PAM VU0422288 to mice modeling the mild R306C mutation (*Mecp2*^*R306C/*+^) and found a significant reduction in apneas. These results provide the first evidence of potentially broad utility for mGlu_7_ PAMs in reducing apneas across the RTT spectrum.

## Introduction

Rett syndrome (RTT) is caused by mutations in the X-linked Methyl-CpG Binding Protein 2 (*MECP2*) gene, a transcriptional regulator of gene expression (Amir et al., 1999; Ben-Shachar et al., 2009; Abbas et al., 2024). RTT patients develop normally during their first 6 to 18 months of life and then undergo a rapid developmental regression, resulting in impairments in language and motor skills and the emergence of breathing abnormalities (such as apneas) and seizures (Neul et al., 2010; Gold et al., 2024; Lopes et al., 2024). Given the monogenic nature of the disease, many therapeutic strategies are aimed specifically at MeCP2 and include gene therapy, DNA and RNA editing, X-chromosome reactivation, and pharmacological read-through approaches (reviewed in (Percy, 2016; Palmieri et al., 2023; Gold et al., 2024; Lopes et al., 2024); NCT05898620, NCT06152237)). Currently, the sole FDA approved symptomatic treatment for RTT is the compound trofinetide, which is a modified version of a glycine-proline-glutamate tripeptide cleaved from insulin growth factor 1 (IGF-1 (Furqan, 2023; Harris, 2023; Neul et al., 2023)), although *MECP2*-based clinical trials for gene therapy have been reported to induce encouraging symptom improvement.

We previously performed expression profiling studies from the motor cortex and cerebellum in autopsy samples from patients diagnosed with RTT (these experiments focused on the *MECP2* truncation mutations R168X, R255X, and R270X) and corresponding age, postmortem interval, and sex-matched controls (Gogliotti et al., 2016; Gogliotti et al., 2017; Gogliotti et al., 2018). These studies identified a number of G protein-coupled receptors (GPCRs) that were altered in RTT patients. One target identified was the mGlu_7_ receptor, which was reduced by approximately 70% in both the cortex and cerebellum of RTT patients (Gogliotti et al., 2017). We then showed that abnormal social, respiratory (e.g., apneas), and cognitive phenotypes could be corrected by administering a positive allosteric modulator (PAM) with mGlu_7_ activity, termed VU0422288, to *Mecp2*^*−/y*^ male and *Mecp2*^+*/−*^ female mice (Jalan-Sakrikar et al., 2014; Gogliotti et al., 2017). mGlu_7_ belongs to the group III mGlu receptors, which also includes the related and widely expressed mGlu_4_ and mGlu_8_ receptors (Niswender and Conn, 2010), and VU0422288 potentiates the activity of all of these receptor isoforms (Jalan-Sakrikar et al., 2014). In our previous work, specific mGlu_7_ antagonism and use of control PAMs with different selectivity profiles were strategies used to validate mGlu_7_ as the target of VU0422288 (Gogliotti et al., 2017).

Many RTT patients have missense mutations in the *MECP2* gene that result in different degrees of clinical severity (Yamashita et al., 2001; Leonard et al., 2003; Weaving et al., 2003; Bebbington et al., 2008; Neul et al., 2008; Renieri et al., 2009; Cuddapah et al., 2014; Townend et al., 2018; Collins and Neul, 2022; Downs et al., 2024). For example, the R133C and R306C mutations, along with late truncating mutations, present with milder symptoms, while early truncating mutations, such as R168X and R255X, result in more severe phenotypes (Yamashita et al., 2001; Leonard et al., 2003; Bebbington et al., 2008; Renieri et al., 2009; Cuddapah et al., 2014). Mutation-specific expression patterns of target genes are important, as they raise the possibility that potentiation of mGlu_7_ could be most effective in patients with more severe mutations and less effective in other populations and could inform inclusion criteria for potential clinical trials. Here, we profiled the expression of mGlu_7_, along with the related mGlu_4_ and mGlu_8_ receptors, in brain samples derived from the temporal cortex from a 14-sample cohort of controls and a 41-sample cohort of RTT patients genotyped for *MECP2* mutations (Gogliotti et al., 2017). We found no significant changes in mGlu_4_ or mGlu_8_ expression but identified significant reductions in mGlu_7_ levels that correlated with mutation severity, with significant reductions in the group with *MECP2* mutations that induce more severe phenotypes.

We then examined a mouse model of RTT modeling the milder clinical mutation, R306C, and found that mGlu_7_ expression levels were not reduced in most brain regions, including the brainstem; the exception was the hippocampus, where we did observe a significant reduction in expression. We then treated *Mecp2*^+*/*+^ and *Mecp2*^*R306C/*+^ female mice with a 30 mg/kg dose of the PAM VU0422288, which was previously shown to be effective in *Mecp2*^+*/−*^ mice in correcting apneas (Gogliotti et al., 2017), and found that VU0422288 still exhibited efficacy in reducing apneas in *Mecp2*^*R306C/*+^ animals without affecting other breathing parameters. These results provide evidence validating the efficacy of mGlu_7_ PAMs in disease models of RTT resulting from mutations that induce milder phenotypes, providing potential wide utility for treating RTT patients with distinct mutations.

## Experimental procedures

### Human brain sample profiling

Human samples were obtained from the National Institutes of Health NeuroBioBank (neurobank.nih.gov) under Public Health Service contract HHSN-271-2013-00030. The tissues were post-mortem and fully de-identified, and as such are classified as exempt from human subject research regulations. The Brodmann Area (BA) 38 (temporal cortex) samples used for this study are shown in Table 1. For the majority of these samples, the *MECP2* mutation was unknown upon receipt, and we performed TOPO TA cloning and Sanger sequencing to identify the *MECP2* mutation for each sample using primers that span the length of and corresponding to the e1 isoform (predominantly expressed in the brain) of the gene. Samples ranked “mutation negative” were those for which an MECP2 mutation was not found by Sanger sequencing followed by whole exome sequencing (WES). Samples marked “undefined genotype” were those for which a mutation was not found by Sanger sequencing but that was not subject to WES.

### Animals

All animals used in the present study were group housed with food and water given ad libitum and maintained on a 12hr light/dark cycle. Animals were cared for in accordance with the National Institutes of Health Guide for the Care and Use of Laboratory Animals. All studies were approved by the Loyola Institutional Animal Care and Use Committee and took place during the light phase. *Mecp2*^*306C/*+^ (B6.129P2 (C)-*Mecp2*^*tm5.1Bird*^/J, stock no. 026847) were obtained from The Jackson Laboratory (Bar Harbor, ME, USA) and maintained on a C57BL/6J background by breeding *Mecp2*^*306C/*+^ with WT C57BL/6J mice (The Jackson Laboratory, stock no. 000664). As a reflection of the predominantly female RTT patient population, female *Mecp2*^*306/*+^ mice were utilized and aged to 28 weeks prior to experiments, an age that is symptomatic within our colony.

### Total RNA preparation and quantitative RT-PCR

As previously reported (Smith et al., 2022), approximately 1 g of the temporal cortex was impact-dissociated under dry ice and then pulverized using mortar and pestle under liquid nitrogen. Total RNA was prepared from 200 mg of tissue using standard Trizol-chloroform methodology. cDNA from 2 μg of total RNA was synthesized using a SuperScript^™^ VILO^™^ cDNA Synthesis Kit (ThermoFisher, cat. no. 11754050). qRT-PCR (CFX96, Bio-Rad, Vanderbilt University Medical Center MCBR Core). 50 ng/9μL cDNA was run in duplicate using Taq-Man^™^ Fast Universal PCR Master Mix (2X), no AmpErase^™^ UNG (Life Technologies, cat. no. 4352042) and Life Technologies gene expression assays for human *GRM4* (Hs00904641_m1), *GRM7* (Hs00990476), *GRM8* (Hs00945353_m1) and glucose 6-phosphate dehydrogenase (*G6PD*, Hs00166169_m1). Ct values for each sample were normalized to *G6PD* expression and analyzed using the delta–delta Ct method as performed in (Gogliotti et al., 2017). Each value was compared to the average delta-Ct value acquired for control human samples and calculated as percent-relative to the average control delta-Ct. Data were excluded if significantly different as assessed via a ROUT test.

### Total and synaptosomal protein preparation and western blotting

The cortex, hippocampus, striatum, cerebellum and brainstem were microdissected from 28-week-old female WT (*Mecp2*^+*/*+^) and *Mecp2*^*306C/*+^ littermates. Synaptosomes were isolated according to a protocol described in (Fentress et al., 2013). Mouse total protein (50 μg) was electrophoretically separated using a 4–20% SDS polyacrylamide gel and transferred onto a nitrocellulose membrane (iBlot2, Thermo-Fisher). Membranes were blocked in TBS Odyssey blocking buffer (LI-COR) for 1hr at room temperature. Membranes were probed with primary antibodies overnight at 4°C: rabbit anti-mGlu_7_ (1:1000, EMD Millipore cat no. 07–239), mouse anti-tubulin (1:5000, Abcam cat. no. ab44928) or mouse anti-Gapdh (1:5000, ThermoFisher/Invitroen, cat. no. MA5–15738), followed by the fluorescent secondary antibodies: goat anti-rabbit (800 nm, 1:5000, LI-COR, cat. no. 926–32211) and goat anti-mouse (680 nm, 1:10,000, LI-COR, cat. no. 926–68020). Fluorescence was detected using the Odyssey (LI-COR) imaging system at the Vanderbilt University Medical Center Molecular Cell Biology Resource (MCBR) Core and then quantified using the Image Studio Lite software (LI-COR). Levels of monomeric and dimeric mGlu_7_ were normalized to tubulin. None of the blots were stripped and reprobed.

### Drugs

VU0422288 was purchased from Tocris Bioscience (Bristol, UK, catalog number 5378).

### Whole body plethysmography

The apnea study was designed to include a control group for genotype (*Mecp2*^+*/*+^ animals) as well as a control for compound (vehicle). This was an acute study performed with N of 5–7 animals per genotype and treatment, animals were not assessed for breathing parameters prior to treatment, were randomized prior to compound administration, and the experimenter was blinded to genotype and treatment. Unrestrained 28-week-old *Mecp2*^+*/*+^ and *Mecp2*^*R306C/*+^ mice were placed in a whole-body plethysmograph recording chamber (Buxco, 2-site system) with a continuous inflow of air (1 L/min). Following a habituation period of 30 min, a baseline recording was established for 30 min. Mice were then removed from the chamber, injected with VU0422288 or vehicle, and reacclimated for 30 min, and respiratory measurements were made for an additional 30 min. Analysis was performed using FinePointe Research Suite (v2.3.1.9). Apneas, defined as pauses spanning 2 × the average expiratory time of the previous 2 min, were quantified using the FinePointe apnea software patch, followed by manual spot-checking of the larger data set. Only periods of motion-free recording were analyzed. All filters were applied while the researchers were blinded to the genotype and treatment group. One animal in the WT vehicle group was ranked as an outlier by ROUT analysis and removed. The final analysis was performed using t-tests between each genotype to compare treatment effects.

### Statistical analysis

Statistics were carried out using GraphPad Prism. All data shown represent mean ± SEM, individual points are shown for each dataset, and the statistical test used for each set of data is described in the respective legend. Normality tests using a Shapiro-Wilk test were performed for all data.

## Results

**Fig. 1.**
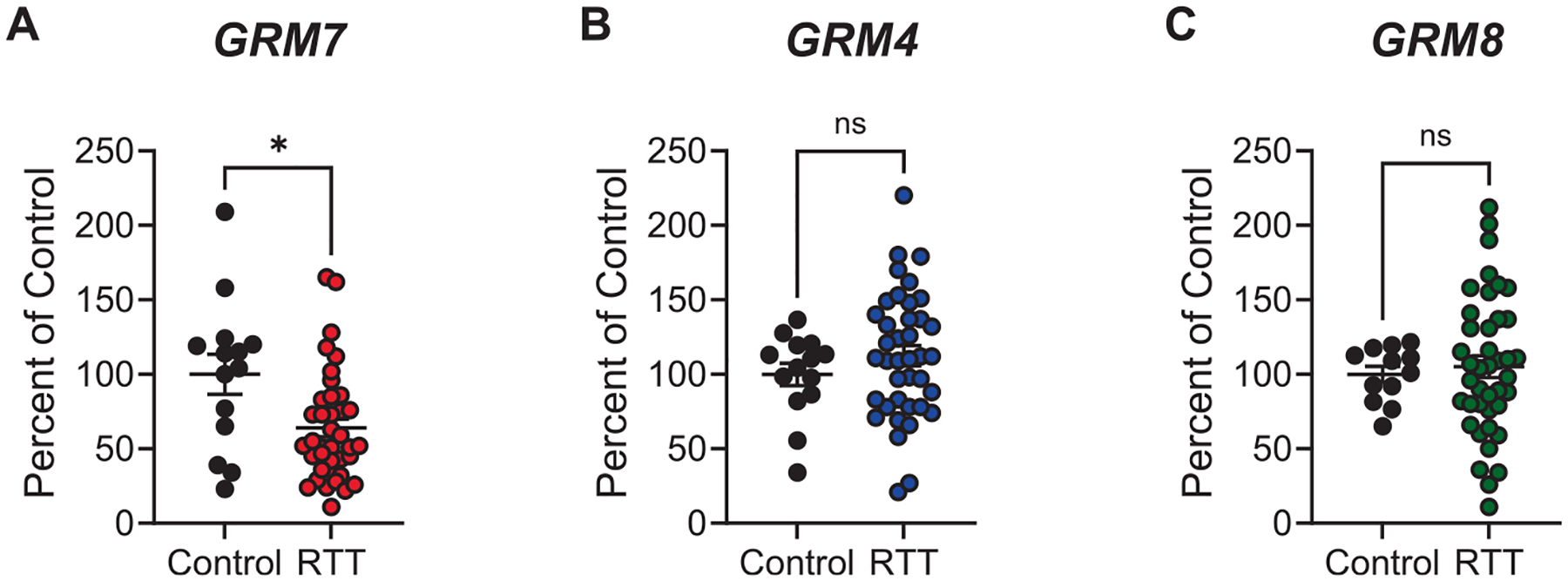
Among the group III mGlu receptors, only *GRM7* mRNA is decreased in cortical autopsy samples from RTT patients. RNA was prepared from human tissue from the BA38 region of the temporal cortex and quantitative RT-PCR was performed for (A) *GRM7*, (B) *GRM4*, and (C) *GRM8*. Data shown are mean ± SEM and were performed in duplicate and averaged and normalized to *G6PD* control. Data normality was tested using a Shapiro-Wilk test. (A) Control: W = 0.9467, p = 0.5113; RTT: W = 0.9085, **p = 0.0045. (B) Control: WT: W = 0.9075, p = 0.1449; RTT: W = 0.9883, p = 0.9519. (C) Control: WT: W = 0.9252, p = 0.3317; RTT: W = 0.9847, p = 0.8450). Data were analyzed using a Welch’s *t*-test for panel (A) (t(18.5) = 2.456, *p = 0.0242) or unpaired t-tests for *GRM4* and *GRM8* (*GRM4*, t(51) = 1.037, p = 0.3047); *GRM8*, t(51) = 0.3646, p = 0.7170). Outliers in each dataset determined using a ROUT test are noted in Table 1.

We previously profiled mGlu_7_ expression using motor cortex and cerebellar samples from seven RTT patients with either R168X, R255X, or R270X truncating mutations in MeCP2 as well as age, brain region, post-mortem interval, and sex matched controls (Gogliotti et al., 2017). These experiments identified a significant reduction in expression of the mGlu_7_ receptor in the cortex and cerebellum of RTT patients (Gogliotti et al., 2017). Here, we expanded these studies to samples from the temporal cortex of a larger, 41 sample cohort of RTT patients representing a range of *MECP2* mutations (Table 1 lists each patient’s mutation, post-mortem interval, and age). These mutations ranged from the methyl binding domain (Y141X, T158M, R168X), to the transcription repression domain (R255X, G269Del, R270X), to the Nuclear hormone receptor Co-Repressors (NCoR) interaction domain/C-terminal domain (P302L, R306C, P322L). Within our cohort, there were five samples for which we did not detect a mutation in *MECP2* by Sanger sequencing followed by whole exome sequencing, but the patient had received a clinical RTT diagnosis (these are listed as “mutation negative”). Eight additional samples were ranked as “undetermined” due to poor sequencing results; these patients had also all received a RTT diagnosis and all patients were included in the analysis in Fig. 1. There was no difference between control and RTT subjects for either age (20.9 ± 2.2 years, control, versus 23.1 ± 2.0 years, RTT; normality tested by Shapiro-Wilk test: control, W = 0.9080, p = 0.1475; RTT, W = 0.9154; **p = 0.0056; Mann-Whitney test, p = 0.6497, Mean ± SEM) or post-mortem interval (19.2 ± 1.7, control, versus 21.0 ± 1.6 h, RTT; normality tested by Shapiro-Wilk test, control, W = 0.8891, p = 0.1096; RTT, W = 0.9769, p = 0.5772; unpaired *t*-test, p = 0.5441, Mean ± SEM).

We performed qRT-PCR to examine *GRM7* mRNA expression (Table 1, Fig. 1) across this cohort of control and RTT patients. Expression values in three RTT samples were ranked as outliers for *GRM7* and these samples were removed from the analysis in Fig. 1 but are noted in Table 1. We observed a statistically significant (*p = 0.0242, Welch’s *t*-test between control and RTT) reduction in *GRM7* mRNA expression across the cohort when these outliers were excluded (Fig. 1A, black versus red, Table 1). We performed similar profiling for mRNA encoding *GRM4* (two outliers excluded) and *GRM8*, the other widely CNS-expressed group III mGlu receptors, and we did not detect significant differences between control and RTT patients for mRNAs encoding these receptors (Fig. 1B, C, Table 1; *GRM4*, p = 0.3047; *GRM8*, p = 0.7170, unpaired *t*-test).

**Fig. 2.**
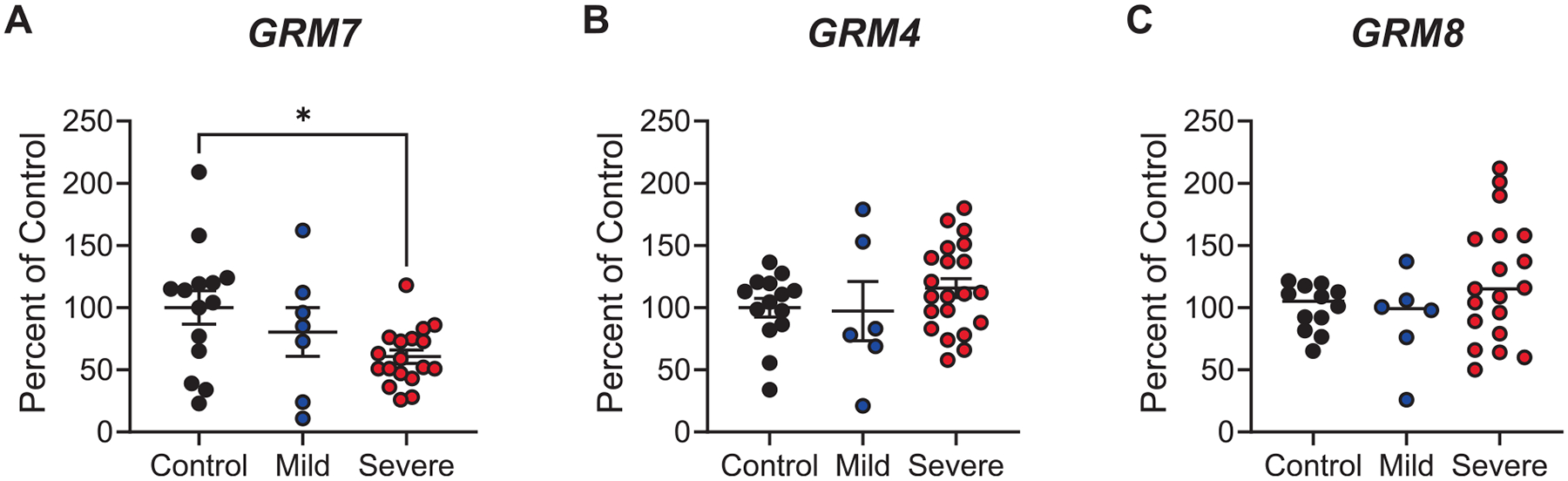
*GRM7* expression is significantly decreased in patients with mutations that induce more severe, but not mild, RTT symptoms. (A) *GRM7* mRNA is significantly reduced in patients with severe (red, Y141X, T158M, R255X, R2760X, R272–273 Del, G269 Del), but not in patients with mild mutations (blue, R133C, R306C, P302L, P322L, L386fs, CTD > 398). (B) *GRM4* and (C) *GRM8* are not significantly different in any group versus control. Data represent mean ± SEM and data normality was tested using a Shapiro-Wilk test and found to be normal for all groups. (A) Control: W = 0.9467, p = 0.5113; Mild: W = 0.9626, p = 0.8407; Severe: W = 0.9524, p = 0.4636. (B) Control: W = 0.9075, p = 0.1449; Mild: W = 0.9294, p = 0.5754; Severe: W = 0.9706, p = 0.7462. (C) Control: W = 0.9252, p = 0.3317; Mild: W = 0.9239, p = 0.5339; Severe: W = 0.9542, p = 0.4652. Data were analyzed using Ordinary One-Way ANOVA: *GRM7*, F (2, 26), 3.829, *p = 0.0366; Dunnett’s post-hoc test, control versus mild, p = 0.4788; control versus severe, *p = 0.0259. *GRM4*, Ordinary One-Way ANOVA, F (2, 38) 1.040, p = 0.3634. *GRM8*, Ordinary One-Way ANOVA, F (2, 34) 1.767, p = 0.1862.

To determine if there was a correlation of group III mGlu mRNA expression with *MECP2* mutation location or disease severity, we next binned samples into mild and severe groups based on previous determinations of severity of a large cohort of RTT patients (Cuddapah et al., 2014). The mild group contained samples corresponding to the mutations R133C, P302L, R306C, L386fs, CTD > 398, and the severe group included samples corresponding to the mutations Y141X, T158M, R167X, R168X, R255X, R269 Del, R270X, and R272–273 Del. There were five RTT samples that had been found to be *MECP2* mutation negative by Sanger sequencing (Table 1). While these samples appeared to be lower in expression compared to control, these samples were not significantly different when assessed using an exact Mann Whitney test (control, 100 ± 13.4 (n = 14) versus mutation negative RTT (n = 5), 52.2 ± 19.8, p = 0.1068); this may be due to the low number of samples and high variability in this group. These studies revealed that, when the control, mild, and severe groups were compared across patients with defined *MECP2* mutations, there were only statistically significant reductions in *GRM7* mRNA in patients with mutations in the severe but not the mild group (Fig. 2A, Table 1). In contrast, we did not observe any statistically significant differences in expression for *GRM4* or *GRM8* that correlated with MeCP2 mutation status (Fig. 2B and C).

**Fig. 3.**
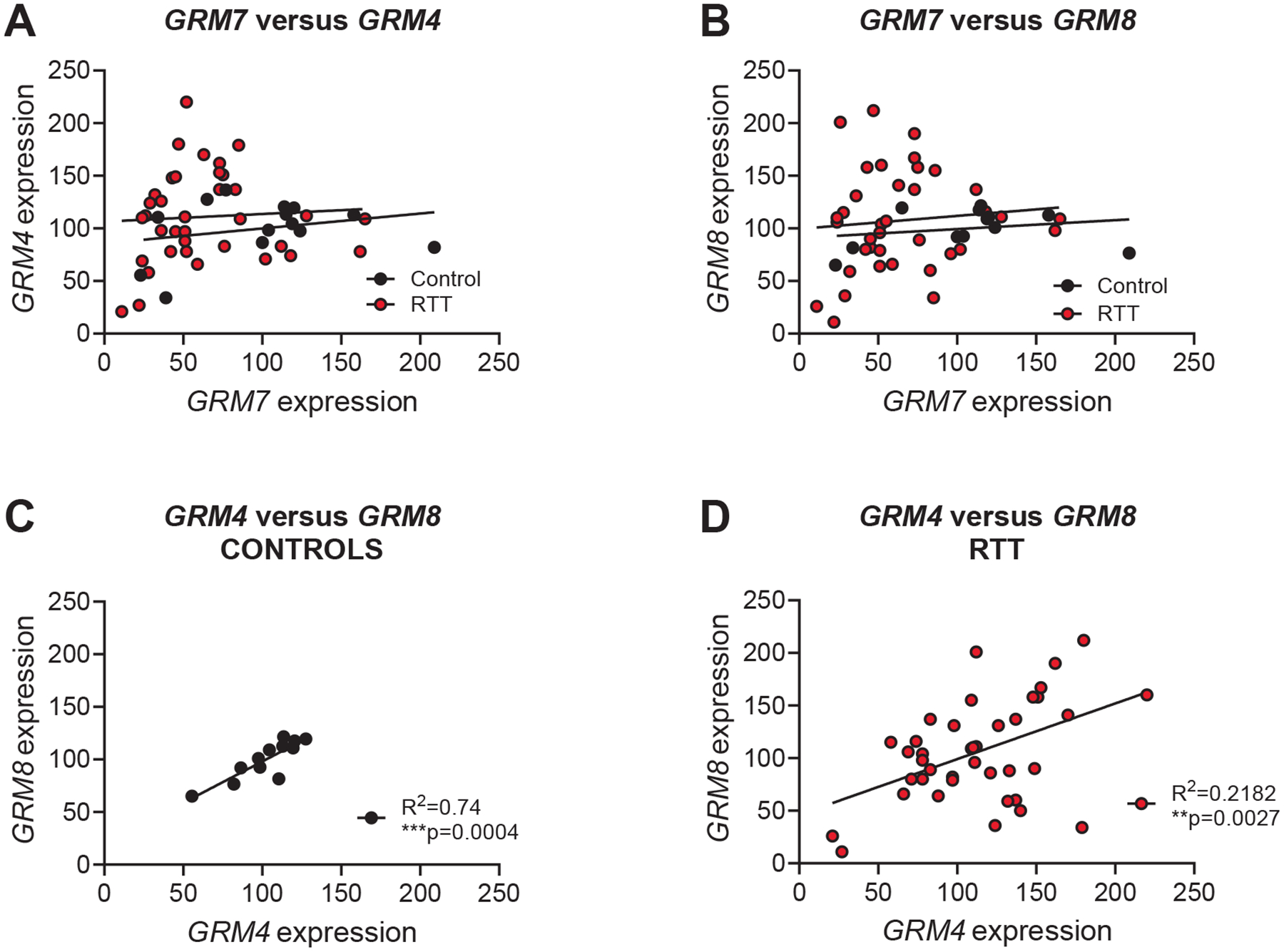
*GRM7* mRNA expression does not correlate with either *GRM4* and *GRM8*; however, *GRM4* and *GRM8* expression exhibits a significant correlation in human cortical tissue. mRNA expression data from the experiments in Table 1, and Fig. 1, were correlated by linear regression for (A) *GRM7* and *GRM4*, (B) *GRM7* and *GRM8*, and *GRM4* and *GRM8* for control (C) and (D) RTT samples. Significant correlations were observed for panels (C) (R^2^ = 0.7352, F = 27.77, ***p = 0.0004) and (D) (R^2^ = 0.28, F = 10.33, ***p = 0.0027).

We then performed correlation analyses to determine if there was co-regulation of *GRM7* with either *GRM4* or *GRM8* in the human cortex (Fig. 3), as the group III mGlu receptors have been shown to heterodimerize (Doumazane et al., 2011; Liu et al., 2017; Moreno Delgado et al., 2017; Xiang et al., 2021; Lin et al., 2022). These studies revealed that there was no correlation between *GRM7* with *GRM4* or *GRM8* expression (Fig. 3A, B). Interestingly, however, there was a highly significant correlation in expression between *GRM4* and *GRM8*, particularly in the control samples (linear regression, R^2^ = 0.7352, F = 27.77, ***p = 0.0004; Fig. 3C) that was also present in the RTT cohort (**p = 0.0027, Fig. 3D). To our knowledge, this expression correlation has not been reported for these two receptors in the human brain. Finally, we performed correlations of *GRM7*, *GRM4*, and *GRM8* with post-mortem interval (PMI) and age of the samples in our cohort. No correlation was established for any of the three transcripts with PMI (data not shown); however, we quantified a negative correlation of *GRM7* and *GRM8* with age in control, but not RTT, samples (*GRM7*, Linear regression, *p = 0.0364; *GRM8*; *p = 0.0223).

**Fig. 4.**
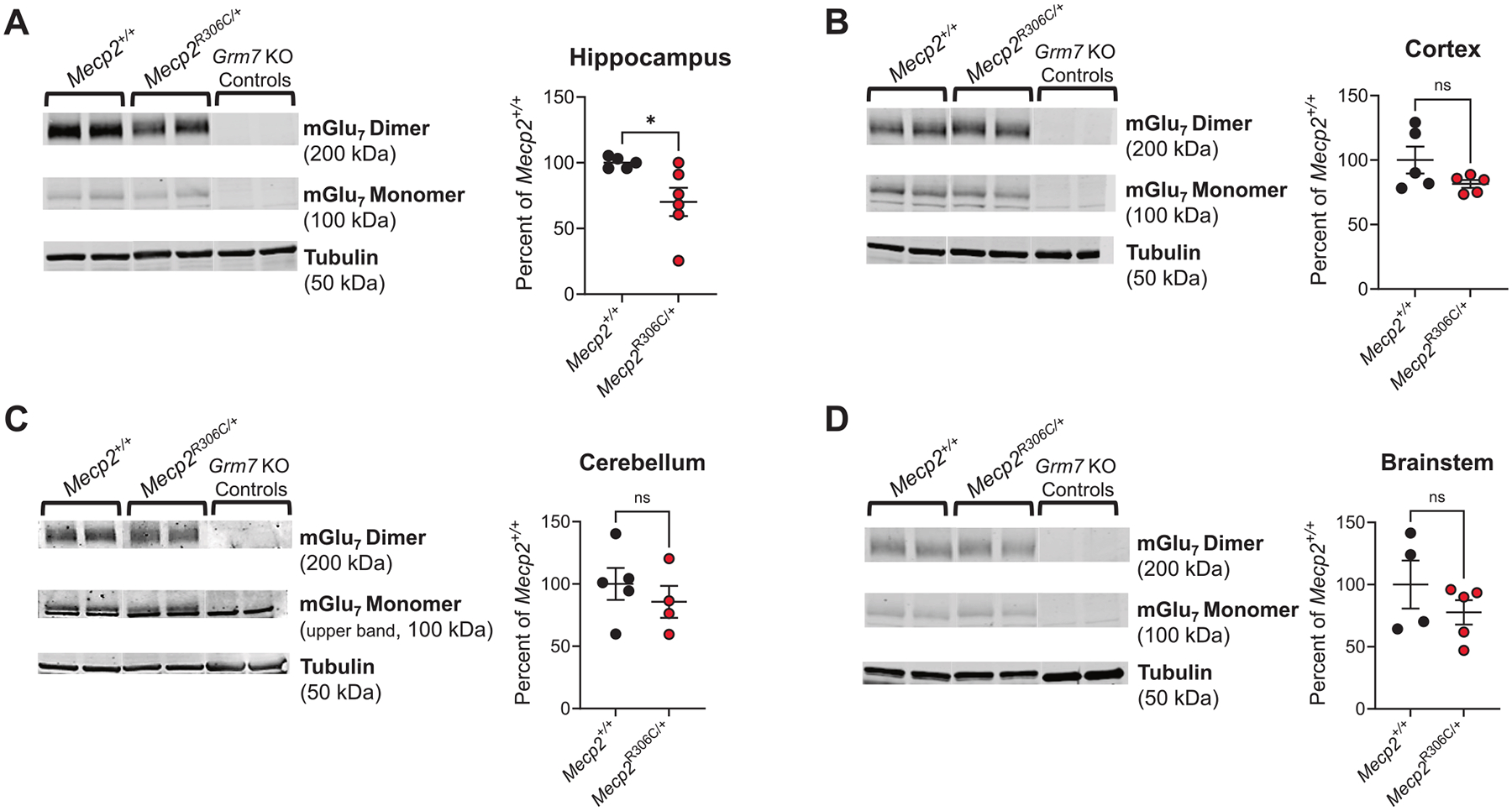
In mice modeling the milder R306C mutation, mGlu_7_ expression in synaptosomes is significantly reduced in the hippocampus, but not the cortex, cerebellum, or brainstem. mGlu_7_ protein levels were assessed in (A) hippocampus, (B) cortex, (C) cerebellum, and (D) brainstem from *Mecp2*^+*/*+^ (WT, black) and *Mecp2*^*R306C/*+^ (RTT, red) female mice. Representative Western blots are shown with the presence of monomeric and dimeric forms of mGlu_7_ as well as mGlu_7_ KO controls (full blots are shown in Supplemental Figs. 1–4). Data represent mean ± SEM and data normality was tested using a Shapiro-Wilk test and found to be a normal distribution for all groups (A) *Mecp2*^+*/*+^: W = 0.8970, p = 0.3936; *Mecp2*^*R306C/*+^: W = 0.9466, p = 0.7123. (B) *Mecp2*^+*/*+^: W = 0.8571, p = 0.2179; *Mecp2*^*R306C/*+^: W = 0.8667, p = 0.2533. (C) *Mecp2*^+*/*+^: W = 0.9492, p = 0.7314; *Mecp2*^*R306C/*+^: W = 0.9579, p = 0.7656. (D) *Mecp2*^+*/*+^: W = 0.8609, p = 0.2635; *Mecp2*^*R306C/*+^: W = 0.8385, p = 0.160). For panel (A), F, 37.84, t(9) = 2.487, *p = 0.0346, by unpaired Student’s *t*-test; all other t-tests were not significant. Full Western blots are shown in Supplemental Figs. 1–4.

**Fig. 5.**
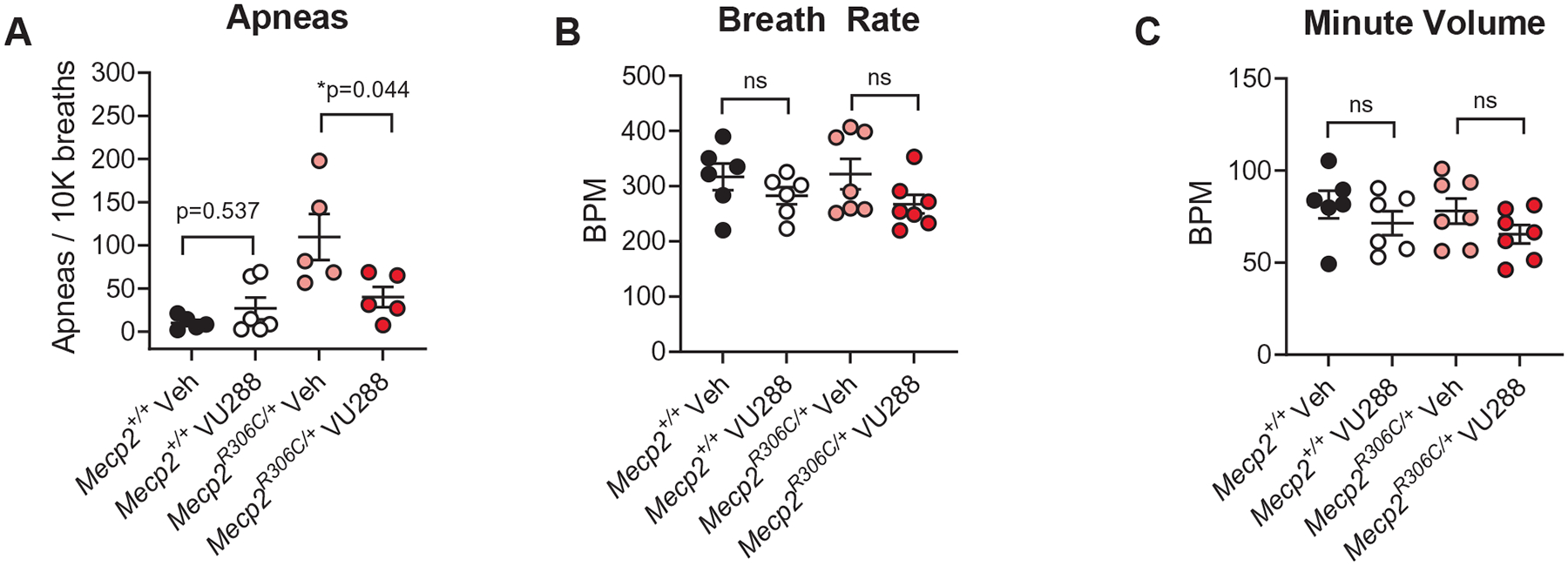
VU0422288 (VU288) reverses apneas in ^*Mecp2 R306C/*+^ mice. (A) Apneas were assessed using whole body plethysmography (WBP) and normalized to 10,000 breaths for *Mecp2*^+*/*+^ (WT, black) and *Mecp2*^*R306C/*+^ (RTT, red) female mice. Data normality was tested using a Shapiro-Wilk test (*Mecp2*^+*/*+^, Veh: W = 0.9611, p = 0.8159; *Mecp2*^+*/*+,^ VU288: W = 0.7598, *p = 0.0248; *Mecp2*^*R306C/*+^, Veh: W = 0.8827, p = 0.3218; *Mecp2*^*R306C/*+^, VU288: W = 0.9022, p = 0.4221). Data between drug treatments (i.e., between vehicle and VU288) were compared via a Mann-Whitney test for WT (p = 0.5368) and an unpaired *t*-test for RTT (F = 5.145, t(8) = 2.391, *p = 0.0438). (B) Breath rate was not different after treatment in either genotype. Data normality was tested using a Shapiro-Wilk test. *Mecp2*^+*/*+^, Veh: W = 0.9642, p = 0.8514; *Mecp2*^+*/*+^, VU288: W = 0.9433, p = 0.6858; RTT, Veh: W = 0.7900, *p = 0.0326; *Mecp2*^*R306C/*+^, VU288: W = 0.9078, p = 0.3806. *Mecp2*^+*/*+^, Veh versus *Mecp2*^+*/*+^, VU288: unpaired *t*-test, t(10) = 1.194, p = 0.2599. *Mecp2*^*306C/*+^ Veh versus VU288: Mann Whitney test, p = 0.1282. (C) Minute volume was not different after treatment in either genotype. Data normality was tested using a Shapiro-Wilk test, *Mecp2*^+*/*+^, Veh: W = 0.9027, p = 0.3904; *Mecp2*^+*/*+^, VU288: W = 0.8794, p = 0.2665; *Mecp2*^*R306C/*+^, Veh: W = 0.9009, p = 0.3367; *Mecp2*^*R306C/*+^, VU288: W = 0.9465, p = 0.6981). *Mecp2*^+*/*+^ Veh versus *Mecp2*^+*/*+^, VU288: unpaired *t*-test, t(10) = 1.026, p = 0.3292. *Mecp2*^*306C/*+^ vehicle versus VU288: unpaired *t*-test, t(12) = 1.491, p = 0.1617.

The observation that reductions in mGlu_7_ were not present in every patient suggests that RTT patients with certain mutations may not respond to a PAM with mGlu_7_ activity. To address this possibility, we chose the R306C mouse model of RTT which represents a milder form of the disease (Cuddapah et al., 2014) and profiled synaptic mGlu_7_ protein expression in multiple brain areas from 28-week-old female mice. Expression studies showed a significant decrease in mGlu_7_ protein levels in the hippocampus but not in other brain areas such as the cortex, cerebellum, or brainstem (Fig. 4, Supplemental Figs. 1–4). Our previous work has shown that VU0422288 robustly reduces apneas in *Mecp2*^+*/−*^ animals as assessed using whole body plethysmography (Gogliotti et al., 2017). In *Mecp2*^*306C/*+^ animals, we also observed a significant apnea reversal when the animals were administered a 30 mg/kg dose of VU0422288 (Fig. 5A), but no change in breath rate or minute volume (Fig. 5B and C). This result was observed despite the normal levels of mGlu_7_ in the cortex and brainstem, suggesting that VU0422288′s efficacy may not be linked solely to target disruption, and suggests that mGlu_7_ PAMs have the potential to retain utility across multiple RTT patient subpopulations in reducing apneas.

## Discussion

The discovery that mutations in MeCP2 cause RTT was made over 25 years ago; however, there is currently only one FDA-approved treatment for this disorder, a 3 amino acid peptide, trofinetide (Collins and Neul, 2022; Harris, 2023; Kennedy et al., 2023; Neul et al., 2023; Abbas et al., 2024; Neul et al., 2024). There is much optimism for trials are now underway for *MECP2*-based gene therapy, with promising initial reports. Additional therapeutic strategies, such as RNA editing, DNA editing, X-chromosome reactivation, read-through therapies, and oligotherapeutics are also intense foci for potential RTT treatment (Collins and Neul, 2022; Palmieri et al., 2023; Lopes et al., 2024; Percy et al., 2024; Vanderplow et al., 2024).

mGlu_7_ is a GPCR that is amenable to drug development. The receptor is expressed pre-synaptically in the active zones of both glutamatergic and GABAergic neurons, where it functions to decrease neurotransmitter release upon stimulation (Shigemoto et al., 1996; Dalezios et al., 2002; Somogyi et al., 2003; Niswender and Conn, 2010; Gee et al., 2014; Klar et al., 2015). We have demonstrated a particularly important role for mGlu_7_ in modulating GABA release in the hippocampus at Schaffer Collateral-CA1 (SC-CA1) synapses, where mGlu_7_ antagonism prevents the induction of long-term potentiation (LTP), the molecular correlate of learning and memory (Klar et al., 2015), and similar effects of mGlu_7_ antagonists on LTP have been shown in the amygdala (Gee et al., 2014). We and others have performed extensive evaluations of *Grm7* knockout (*Grm7*^*−/−*^) mice, and they exhibit significant impairments in cognition, learning, and response to stimulants. Additionally, they develop spontaneous seizures and show abnormal repetitive movements (Sansig et al., 2001; Bushell et al., 2002; Fisher et al., 2020). Administration of a compound that activates mGlu_7_ prevents both seizures in mice and the development of epilepsy in a kindling model (Girard et al., 2019), while mGlu_7_ negative allosteric modulators (NAMs) and mice encoding a modified receptor C-terminus to prevent protein/protein interactions with Protein Interacting with C-Kinase 1 (PICK1) exhibit seizures (Bertaso et al., 2008; Tassin et al., 2016), suggesting that increasing mGlu_7_ activity might serve as a novel therapeutic approach for epilepsy-related disorders. Recent reports have also shown that people with loss-of-functions mutations in *GRM7* exhibit neurodevelopmental symptoms such as seizures, impaired myelination, severe intellectual disability, apneas, and stereotypies (Charng et al., 2016; Reuter et al., 2017; Fisher et al., 2018; Marafi et al., 2020; Fisher et al., 2021; Song et al., 2021). Combined, these studies suggest that low levels of mGlu_7_ expression are deleterious in numerous neurological domains.

We previously profiled mGlu_7_ mRNA and protein expression in human brain samples and showed that RTT patients with truncating mutations in *MECP2* expressed 60–70% lower levels of mGlu_7_ protein in the cortex and cerebellum (Gogliotti et al., 2017). Using a small molecule that potentiates mGlu_7_, VU0422288, we found numerous benefits in both *Mecp2*^*−/y*^ and *Mecp2*^+*/−*^ animals, including improvements in hippocampal LTP, cognition, social interaction, and breathing abnormalities such as apneas (Gogliotti et al., 2017). While this initial study suggested that mGlu_7_-targeted therapy could be a promising new approach, the clinical landscape of RTT is heterogeneous, and severity is dependent upon the specific MeCP2 mutation found in an individual patient as well as other factors such as X-chromosome inactivation status (Neul et al., 2008; Cuddapah et al., 2014; Gold et al., 2024; Percy et al., 2024). For example, the R133C and R306C mutations are generally milder in disease severity (Yamashita et al., 2001; Leonard et al., 2003; Neul et al., 2008; Gold et al., 2024; Percy et al., 2024). This reality suggests that trials for RTT with any drug, or, in this case, an mGlu_7_ PAM, may need to take into consideration patient mutation as a potential modifier of treatment efficacy.

Our original mGlu_7_ profiling work was limited to seven patients, and all had truncating mutations that are associated with severe disease (Gogliotti et al., 2017). For the current study, we obtained additional samples from patients with a range of RTT mutations to profile the expression of *GRM7* (Table 1, (Smith et al., 2022)). Additionally, although we previously showed that the activity of the group III mGlu receptor PAM VU0422288 was mediated by mGlu_7_ in an *Mecp2*^+*/−*^ model using pharmacological tools to exclude mGlu_4_ and mGlu_8_, we also profiled the expression of *GRM4* and *GRM8* across this wider cohort. These studies revealed that, among the three widely expressed group III mGlu receptors, only *GRM7* mRNA was significantly reduced. This current work expands upon our previous studies to show that mGlu_7_ expression at the mRNA level is significantly reduced in RTT patients. In our previous report (Gogliotti et al., 2017), we did not achieve significance in mRNA reduction in the subset of RTT patients examined; therefore, this current work provides the first evidence in humans that mGlu_7_ levels are reduced at the level of transcription or mRNA stabilization in RTT patients. These findings may suggest that the *GRM7* gene is transcriptionally regulated by MeCP2, although additional studies will be needed to validate this hypothesis. We would note that there were three samples that were ranked as outliers via a ROUT test for mGlu_7_ expression (Table 1, starred), and we currently do not know why these samples are outliers as they span several different mutations (R255X, M246Del, and undefined, Table 1). Currently, we do not have an explanation for these differences in *GRM7* mRNA expression for these specific RTT patients, although X-chromosome inactivation status or medication usage may contribute, as well as other *MECP2*-dependent and independent modifiers. Additionally, we did not observe a correlation in expression of any group III receptor with PMI, and, although we did observe significant negative correlations of *GRM7* and *GRM8* expression with age in the control group, this was not the case for RTT.

Interestingly, while we did not establish a correlation to suggest co-regulation of *GRM7* with *GRM4* or *GRM8*, we found a very strong correlation of the expression of *GRM4* and *GRM8* in both control and RTT groups (Fig. 3). This finding is intriguing from the standpoint of mGlu receptor heterodimerization. While the mGlu receptors function as constitutive dimers, they have recently been found to heterodimerize (Doumazane et al., 2011; Yin et al., 2014; Levitz et al., 2016; Moreno Delgado et al., 2017; Habrian et al., 2019; Lee et al., 2020; Xiang et al., 2021; Lin et al., 2022; Kukaj et al., 2023; Lin et al., 2024). mGlu_4, 7 and 8_ all belong to the group III mGlu receptor subfamily and can heterodimerize among each other as well as with the group II mGlu receptors, mGlu_2_ and mGlu_3_ (Doumazane et al., 2011; Lee et al., 2020). mGlu receptor heterodimers represent a fascinating pharmacological challenge and opportunity, as drug development for the receptors has, thus far, proceeded with homodimer-expressing systems and emerging evidence indicates that heterodimers exhibit dramatically different pharmacology compared to homodimers (Yin et al., 2014; Liu et al., 2017; Moreno Delgado et al., 2017; Lee et al., 2020; Xiang et al., 2021; Lin et al., 2022; Lin et al., 2024). The observation that *GRM4* and *GRM8* may be co-regulated at the mRNA level in the human brain suggests that this receptor combination may be particularly important from a heterodimer standpoint. Additional co-localization studies will surely shed light on this interesting and robust correlation, and single cell studies will be needed to understand if this correlation reflects co-expression in individual cells, such as neurons, or among cells of different types.

When RTT samples were binned into “severe” and “mild” *MECP2* mutations, we found that the cohort of patients with mutations causing more severe symptoms exhibited significant reductions in *GRM7* mRNA and protein compared to patients with historically mild mutations. We would also note that there may be a decrease in expression in patients exhibiting RTT symptoms but for whom a mutation in *MECP2* is not detected; as these samples represent a very small number of RTT patients, this cohort was extremely small and variable and additional studies would be needed to make a correlation. There are several other disorders that exhibit strong phenotypic overlap with RTT, and patients are often diagnosed with RTT until the causative gene is identified. Some of these other “RTT-like” genes including Transcription Factor 4 ((*TCF4*, the causative gene for Pitt Hopkins Syndrome) (Wirgenes et al., 2012; Sweatt, 2013; Kennedy et al., 2016)), Cyclin-Dependent protein Kinase Like 5 ((*CDKL5*), which causes *CDKL5* Deficiency disorder (Szafranski et al., 2015; Leonard et al., 2022)), and Forkhead box G1 ((*FOXG1*), which causes *FOXG1* syndrome (Brockmann and Staudt, 1993; Mencarelli et al., 2010; Akol et al., 2022; D’Mello, 2023)). We are currently performing studies in a *Tcf4*^+*/−*^ model, including examining mGlu_7_ expression and assessing the therapeutic potential of PAMs, which we anticipate will shed light on the utility of mGlu_7_ potentiation in other disorders related to RTT.

Our correlations of mGlu_7_ expression with mutations that induce higher levels of clinical severity suggested that it was possible that our previous findings of efficacy of VU0422288 in *Mecp2*^+*/−*^ mice could be limited to the context of specific *MECP2* mutations. To test this hypothesis, we profiled mGlu_7_ protein expression in R306C mice. We chose this model as the R306C mutation in RTT patients results in a syndrome with “milder” phenotypes and it was represented in our human cohort. Additionally, Brown et al. created an allelic series of three different point mutations in mice, T158M, R306C, and R133C (Brown et al., 2016), which represent decreasing severity of phenotypes in clinical populations (Neul et al., 2008; Cuddapah et al., 2014) and showed that these mouse models mimicked the human severity levels. Phenotypes in R306C mice were in the middle of the range spanned by T158M and R133C and R306C represents a mutation found in approximately 10% of the RTT population (Neul et al., 2008). This suggested that evaluation of drug effects in mice modeling this mutation would likely have clinical impact for a significant number of patients and, if representative of “mild” disease, could also extend to other mutations. These studies showed that mGlu_7_ was not reduced in synaptosomes prepared from the cortex, cerebellum, or brainstem of these animals compared to controls. In contrast, mGlu_7_ levels were significantly reduced in hippocampal synaptosomes from *Mecp2*^*R306C/*+^ mice. This may suggest that the effects of *Mecp2* loss or mutation are brain region- or cell type-specific as it relates to mGlu_7_ expression, and our human studies in the cortex may not reflect changes in other brain areas such as the hippocampus. The lack of decrease in the brainstem and cortex suggested that it was possible that *Mecp2*^*R306C/*+^ mice might not respond to VU0422288 in terms of apneas, a robust phenotype we had observed was responsive to VU0422288 in *Mecp2*^+*/−*^ animals (Gogliotti et al., 2018). Encouragingly, we did find a significant reduction in apneas when *Mecp2*^*R306C/*+^ animals were dosed with VU0422288. We would note that the current studies do not allow us to determine the brain area or neuronal circuit mediating the efficacy induced by VU0422288, especially as we did not find decreases in mGlu_7_ expression in the brainstem. However, the hippocampus has both direct and indirect input to the brainstem; this is consistent with our previous findings that normalizing activity of brain regions that project, either directly or indirectly, to the brainstem correlates with apnea rescue (Smith et al., 2025). DREADD activation of the prefrontal cortex also rescues apneas in RTT via long-range projections to the brainstem (Howell et al., 2017), and the hippocampus can impact respiratory rhythm by accessing the prefrontal-thalamic network (Basha et al., 2023). As the R306C model is not as well characterized as *Mecp2*^+*/−*^, our future plans include using conditional alleles of mGlu_7_ knockouts and overexpressing mice (e.g., (Freitas et al., 2025)), as well as Cre-regulatable viruses, to restrict expression/knockout of mGlu_7_ to specific neuronal populations in *Mecp2*^+*/−*^ animals, which will provide information that can then be back-modeled onto mice expressing the R306C mutation.

Other limitations to the current study include the need to assess additional point mutant models of RTT representing mild disease, such as R133C (Leonard et al., 2003; Cuddapah et al., 2014). In the one patient here with this mutation, we did not observe decreases in mGlu_7_ expression. Future studies may benefit from a systematic evaluation of several different disease mutations. Also, we did not extensively profile VU0422288 activity in cognitive or social domains in this study in *Mecp2*^*R306C/*+^ mice as we have done previously with other *Mecp2* mutant animals (Gogliotti et al., 2017); these are experiments that would be important extensions of the current work. Additionally, it is not clear that the measurement of mGlu_7_ expression in just the temporal cortex will be reflective of mGlu_7_ expression across the human brain or at the level of the synapse. Profiling of protein expression throughout the brain with a comparison between control and RTT sections may be particularly informative in determining mechanisms by which VU0422288 mediates the reduction in apneas observed. Development of a radio-ligand or PET ligand specific to the mGlu_7_ receptor might be very informative in this regard. While these are important next steps, the current dataset is promising for the hypothesis that mGlu_7_ potentiation will exhibit efficacy in decreasing apneas in RTT patients with mild disease.

## Conclusions

Overall, our results demonstrate a correlation of *GRM7* expression with *MECP2* mutation, and patients with mutations that cause more severe disease express lower amounts of *GRM7* mRNA. We did not observe changes in expression of the related *GRM4* and *GRM8* genes, although expression of these two transcripts was highly correlated in the cortical samples used here. Administration of a PAM with mGlu_7_ activity in a “milder” model of RTT revealed significant efficacy in the reduction of apneas. These results suggest that there is therapeutic potential for mGlu_7_ PAMs in RTT patients with both truncating and point mutations.

## Supplementary Material

1

## Figures and Tables

**Table 1:** Demographics and group III mGlu receptor mRNA expression for human patient samples. mRNA assessments were performed in duplicate and normalized to *G6PD* expression.

Controls	NeuroBioBank Number	Age	PMI	*MECP2*Mutation	*GRM7* mRNA	*GRM4* mRNA	*GRM8* mRNA
CTL-1	1038	24	7	N/A	65	128	120
CTL-3	5161	10	22	N/A	158	113	113
CTL-4	5554	13	15	N/A	119	105	109
CTL-6	5844	42	12	N/A	23	56	65
CTL-7	5566	15	23	N/A	115	114	122
CTL-8	5309	14	8	N/A	120	120	111
CTL-9	4330	19	19	N/A	209	82	77
CTL-10	5446	18	18	N/A	114	121	118
CTL-11	5538	19	24	N/A	104	99	93
CTL-12	6544	30	23	N/A	124	98	101
CTL-13	5646	21	23	N/A	100	87	92
CTL-14	5669	25	29	N/A	77	137	
CTL-15	5670	17	22	N/A	39	34	
CTL-16	5751	25	24	N/A	34	111	82
AVE		**20.9**	**19.2**		**100**	**100**	**100**
SD		**8.2**	**6.5**		**50.2**	**28.0**	**18.5**
SEM		**2.2**	**1.7**		**13.4**	**7.5**	**5.3**
RTT	NeuroBioBank Number	Age	PMI	*MECP2*Mutation	*GRM7* mRNA	*GRM4* mRNA	*GRM8* mRNA
Rett-1	AN19242	41		R255X	75	151	158
Rett-3	AN15579	11	9.6	R255X	43	148	158
Rett-4	AN04573	12	5.0	R270X	165	109	109
Rett-5	AN08016	8	2.9	R255X	86	109	155
Rett-6	AN02091	24	15.8	R255X	36	98	131
Rett-7	AN04121	10	23.5	R270X	83	137	60
Rett-8	AN01182	23	10.5	Undefined	1481*	133	88
Rett-11	AN01508	26	28.1	R306C	96	618*	76
Rett-12	AN18782	28	33.4	Undefined	45	97	82
Rett-13	AN13266	33	23.7	R270X	76	83	89
Rett-14	AN17730	22	31.8	P302L	24	69	106
Rett-15	AN05282	22	18.1	Mut neg	128	112	111
Rett-16	AN15264	35	25.4	R168X	52	78	104
Rett-17	AN00811	31	30.0	Mut neg	32	132	59
Rett-18	AN15541	28	12.1	Undefined	102	71	80
Rett-19	AN07799	25	31.0	T158M	63	170	141
Rett-20	AN13274	26	38.5	Undefined	45	149	90
Rett-21	AN06847	24	24.6	T158M	51	97	79
Rett-22	AN05652	41	48.8	R133C	162	78	98
Rett-23	AN07309	12	16.8	R255X	204*	140	50
Rett-24	AN05180	57	16.7	CTD > 398	112	83	137
Rett-25	AN01082	7	33.2	Y141X	118	74	116
Rett-26	AN17849	12	3.1	R168X	73	137	137
Rett-27	AN14876	26	17.1	272–273 del	28	58	115
Rett-28	AN05375	20	23.3	G269 del	51	88	64
Rett-29	AN07094	18	4.4	Undefined	29	124	36
Rett-30	AN13427	33	17.0	L386fs	11	21	26
Rett-31	AN15255	62	24.0	Undefined	42	78	80
Rett-32	AN05266	21	19.7	R167X	59	66	66
Rett-33	AN09497	27	32.1	R255X	73	162	190
Rett-34	AN19272	16	27.6	Mut neg	22	27	11
Rett-35	AN02949	15	21.3	T158M	47	180	212
Rett-36	AN01220	10	24.0	M246del	479*	121	86
Rett-37	AN05357	12	8.1	R168X	26	112	201
Rett-38	AN03862	35	32.5	G269del	51	111	96
Rett-39	AN06744	29	17.0	Mut neg	24	110	110
Rett-41	AN12551	20	14.1	Mut neg	55	398*	107
Rett-43	AN08774	6	23.5	P322L	73	153	167
Rett-45	AN07559	8	22.7	R306C	85	179	34
Rett-46	AN01325	8	12	Undefined	36	126	131
Rett-48	8870			Undefined	52	220	160
AVE		**23.1**	**21.0**		**64.0**	**112.6**	**105.0**
SD		**12.9**	**10.2**		**37.0**	**42.2**	**46.8**
SEM		**2.0**	**1.6**		**6.0**	**6.8**	**7.3**

PMI: postmortem interval. mGlu_7_ dimer and monomer bands were quantified for each sample.

* These samples are outliers by ROUT analysis. All nonstarred samples were used in Fig. 1 regardless of genotype. Samples marked as “undefined” were negative for *MECP2* mutation via Sanger sequencing but were not confirmed using whole exome sequencing and were excluded from the data shown in Fig. 2. Samples marked “mutation negative” were confirmed by whole exome sequencing to lack mutations in *MECP2*.

## Data Availability

All mentioned data are presented in this published article or the Supplemental Information or are available from the corresponding author upon reasonable request.

## Appendix A. Supplementary data

Supplementary data to this article can be found online at https://doi.org/10.1016/j.neuroscience.2026.01.040.

## Abbreviations:

mGlu_7_: metabotropic glutamate receptor 7
*GRM7*: metabotropic glutamate receptor 7 human gene
RTT: Rett syndrome
*MECP2*: *Methyl-CpG Binding Protein 2*
PAM: positive allosteric modulator
NAM: negative allosteric modulator
mGlu: metabotropic glutamate receptors
GPCRs: G protein-coupled receptors
IGF-1: insulin growth factor 1
*CDKL5*: Cyclin-Dependent Protein Kinase Like 5
*FOXG1*: Forkhead Box G1
