# Supplementary material for: Profiling metabotropic glutamate receptor 7 expression in Rett syndrome: consequences for pharmacotherapy
Source: Neuroscience. Author manuscript; Available in PMC 2026 Jul 2. (PMC13327099; doi:10.1016/j.neuroscience.2026.01.040)
Supplement: 1 [file NIHMS2190219-supplement-1.pptx]

## Slide 1
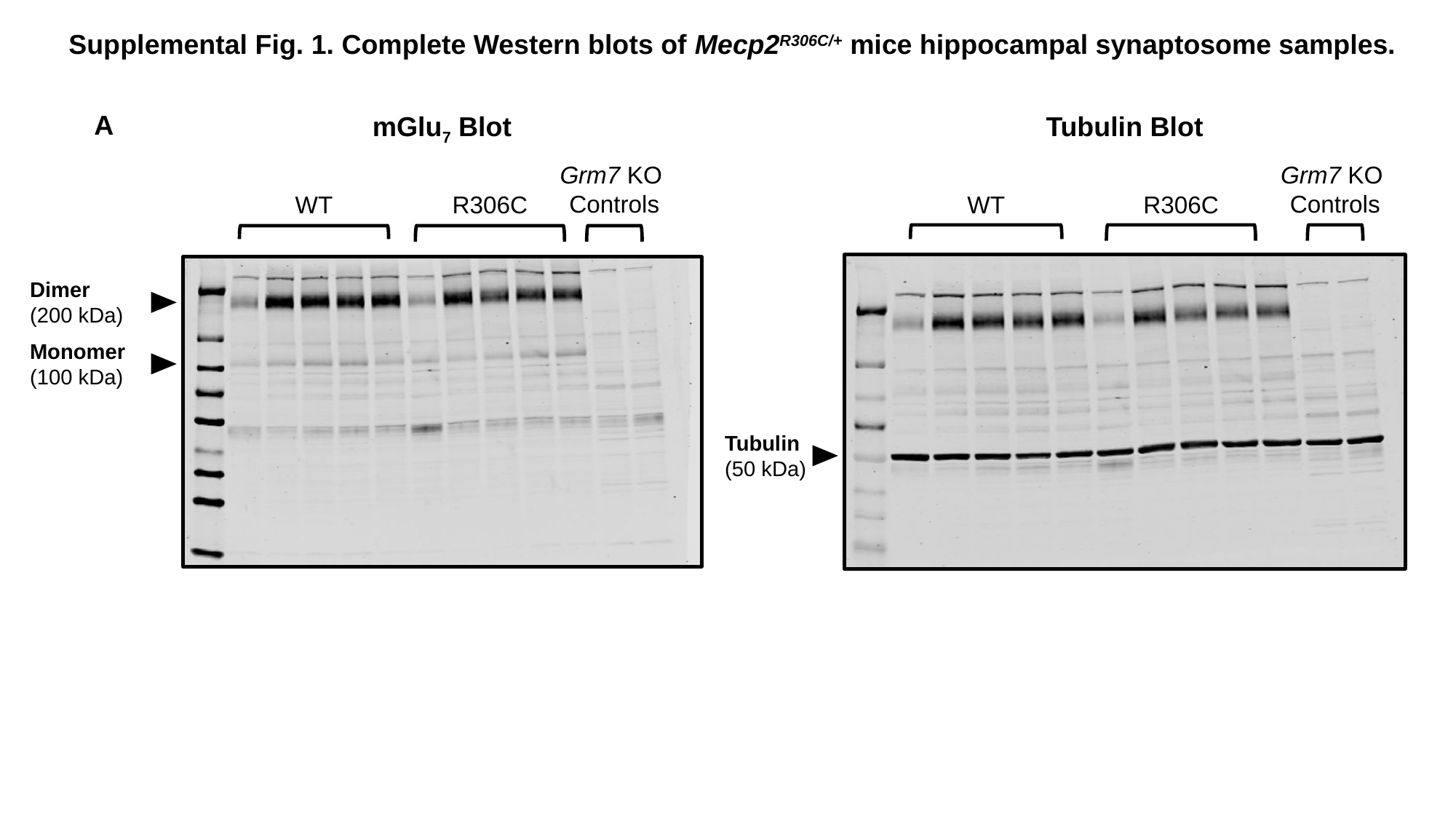

Supplemental Fig. 1. Complete Western blots of Mecp2R306C/+ mice hippocampal synaptosome samples.
A
mGlu7 Blot
Tubulin Blot
Grm7 KO
Controls
Grm7 KO
Controls
WT
R306C
WT
R306C
Dimer
(200 kDa)
Monomer
(100 kDa)
Tubulin
(50 kDa)

## Slide 2
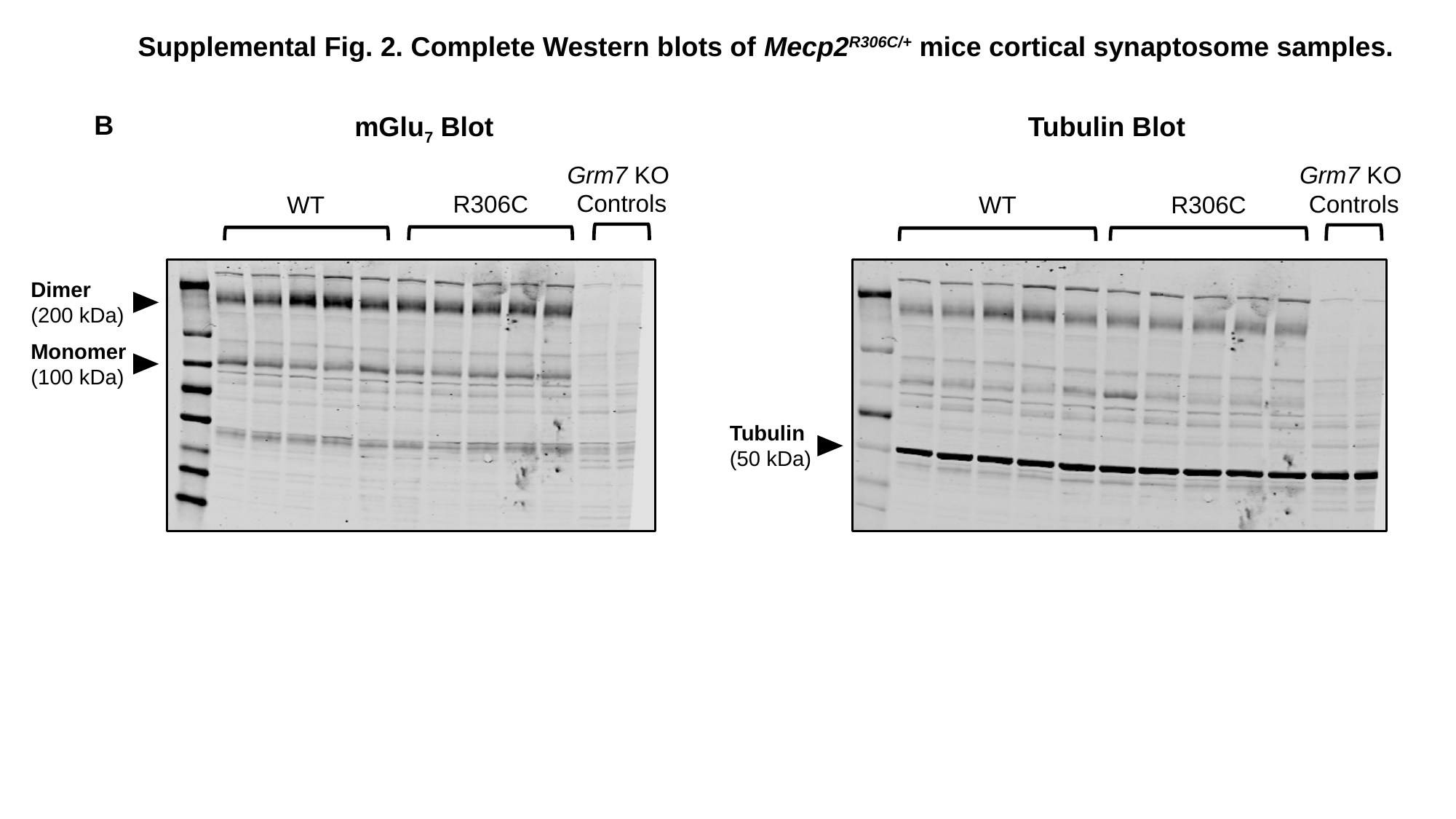

Supplemental Fig. 2. Complete Western blots of Mecp2R306C/+ mice cortical synaptosome samples.
B
mGlu7 Blot
Tubulin Blot
Grm7 KO
Controls
Grm7 KO
Controls
R306C
WT
R306C
WT
Dimer
(200 kDa)
Monomer
(100 kDa)
Tubulin
(50 kDa)

## Slide 3
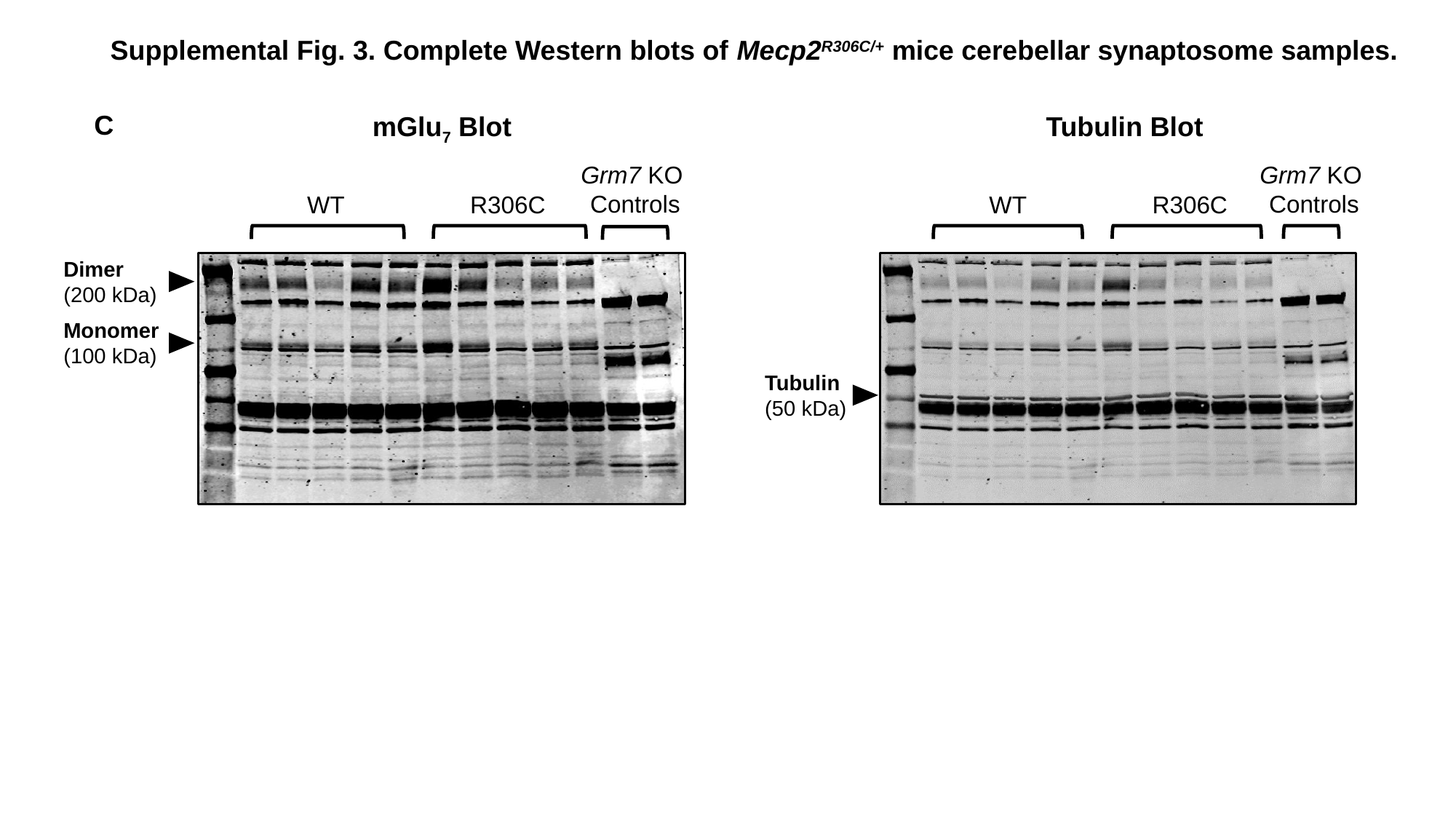

Supplemental Fig. 3. Complete Western blots of Mecp2R306C/+ mice cerebellar synaptosome samples.
C
mGlu7 Blot
Tubulin Blot
Grm7 KO
Controls
Grm7 KO
Controls
WT
R306C
WT
R306C
Dimer
(200 kDa)
Monomer
(100 kDa)
Tubulin
(50 kDa)

## Slide 4
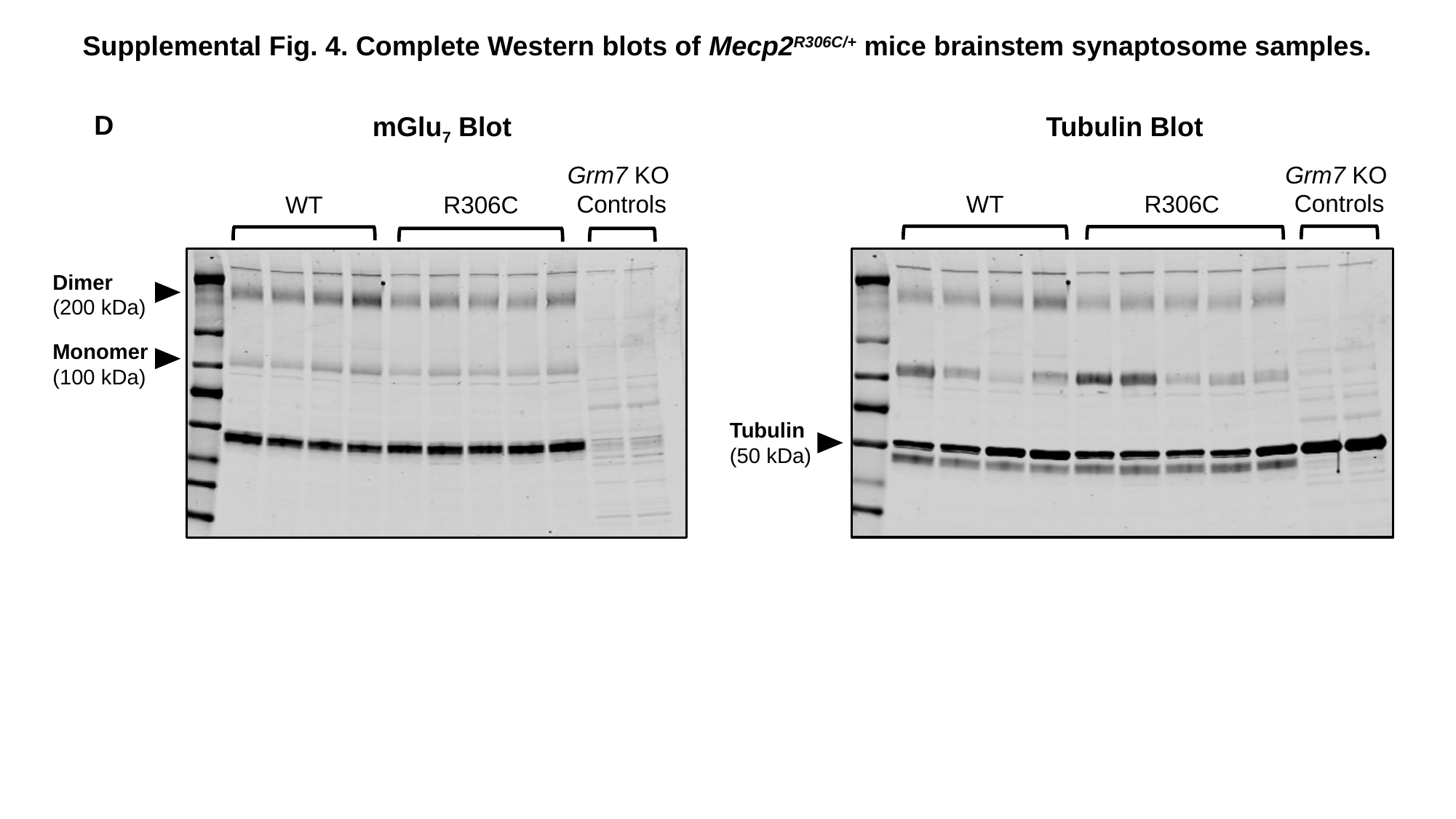

Supplemental Fig. 4. Complete Western blots of Mecp2R306C/+ mice brainstem synaptosome samples.
D
mGlu7 Blot
Tubulin Blot
Grm7 KO
Controls
Grm7 KO
Controls
WT
R306C
WT
R306C
Dimer
(200 kDa)
Monomer
(100 kDa)
Tubulin
(50 kDa)
